# Efficient functional screening of a cellular cDNA library to identify severe fever with thrombocytopenia syndrome virus entry factors

**DOI:** 10.1038/s41598-020-62876-1

**Published:** 2020-04-07

**Authors:** Masayuki Shimojima, Satoko Sugimoto, Satoshi Taniguchi, Tomoki Yoshikawa, Takeshi Kurosu, Masayuki Saijo

**Affiliations:** 1 Special Pathogens Laboratory, Department of Virology I, National Institute of Infectious Diseases, Musashimurayama, Tokyo, Japan; 2 Cooperative Division of Veterinary Sciences, Graduate School of Agriculture, Tokyo University of Agriculture and Technology, Fuchu, Tokyo Japan

**Keywords:** Cellular microbiology, Virus-host interactions

## Abstract

The identification of host cell factors for virus entry is useful for the molecular explanation of viral tropisms and often leads to a more profound understanding of virus-induced diseases. Severe fever with thrombocytopenia syndrome (SFTS) is an emerging infectious disease caused by SFTS virus. No countermeasures against the disease exist. In this report, we show an efficient method using virus-like particles for the functional screening of a cellular cDNA library to identify SFTS virus entry factors. Two variants encoding dendritic cell-specific ICAM-3 grabbing non-integrin related (DC-SIGNR), a calcium-dependent lectin known to enhance SFTS virus infection, were successfully identified from a human liver cDNA library. We will discuss applications for yet unidentified factor(s) for SFTS virus entry and for entry factor(s) for other viruses related to SFTS virus.

## Introduction

Among different organisms, many similar but non-identical cellular molecules exist that show the same functions. Even in a single species, the expressed molecules are dependent on cell types. This is the case with virus entry factors, which are cellular components involved in viral attachment to cells and invasion into cells for viral replication^1,2^. Thus, viral infections show specificity with regards to host ranges, tissues, and cell types, indicating that the identification of virus entry factors is useful for the molecular explanation of viral tropisms^3^. Because target tissues or cell types of viruses are strongly associated with virus-induced diseases, the identification of virus entry factors often leads to a more profound understanding of the associated diseases. Furthermore, the identification of viral entry factors might aid the efficient development of countermeasures against the induced diseases^4,5^.

Severe fever with thrombocytopenia syndrome (SFTS) is a recently recognised tick-borne infectious disease with a high case fatality rate of up to 30%. SFTS virus, whose species name is *Huaiyangshan banyangvirus* (see below for details), is the causative agent^6,7^. There are no specific countermeasures against the disease. In SFTS patients, thrombocytopenia and leukopenia are frequently observed and viral antigens are often detected in the lymphoid organs in fatal cases^8–11^. *In vitro*, most lymphoid cell lines (*e.g*., Jurkat and Raji cells, which originate from T and B cells, respectively) show little susceptibility to SFTS virus infection. In contrast, most non-lymphoid cell lines (*e.g*., Vero cells, which originate from the kidney epithelium) show high susceptibility to the viral infection^8,12–14^. Calcium-dependent (C-type) lectins have been shown to enhance SFTS virus infection when expressed in cells that otherwise show little susceptibility^13,14^; however, this cannot fully explain the viral tropisms because most non-lymphoid cell lines do not generally express them. Nonmuscle myosin heavy chain IIA (NMMHC-IIA) has been reported as a key entry factor of SFTS virus^15^; however, the molecule is expressed even in Jurkat cells and direct interaction with SFTS virus has not been demonstrated. Recent studies reported important roles of glucosylceramide and sorting nexin 11 (SNX11) in the intracellular trafficking and/or penetrating process of SFTS virus infection^16,17^; neither of their expression is cell type-specific and therefore can explain SFTS virus tropisms. These findings indicate that yet unidentified molecules or factors for virus entry contribute to SFTS viral tropism as well as the pathogenesis of SFTS, rather than C-type lectins, NMMHC-IIA, glucosylceramide, and SNX11.

The identification of virus entry factors has been performed by various methods, which are roughly classified into three categories: (I) speculation based on knowledge obtained from experiments^18–21^; (II) screening of libraries based on gain-of-function or loss-of-function criteria^22–24^; and (III) identification of interactive cellular molecules by peptide sequencing or mass spectrometry^25–27^. We have developed an efficient, low-cost cellular cDNA library screening method using a classical panning to identify virus entry factors (hereafter referred to as 1^st^ generation panning for virus entry factor identification or simply 1^st^ generation panning)^28–30^, which could be classified into category II. In 1^st^ generation panning, cellular cDNA libraries are stably expressed in non-adherent cell lines by using a retroviral vector, which integrates genes into cellular genomes with no or low cytotoxicity and do not inhibit cell growth, and screened based on colony formation of target cells on viral particle-coated dishes. From genome of colony-forming cells, cDNAs encoding molecules which confer adhesiveness onto viral particles to non-adherent cells are recovered by polymerase chain reaction (PCR) with primers targeting retroviral vector sequences flanking to cDNA library. Therefore, interaction between viral particles and surface molecules of target cells that is strong enough to trap target cells onto viral particle-coated dishes is a prerequisite for the method. The method was also applied to identify a receptor of a protozoa *Plasmodium falciparum*^31^. For viruses for which the interaction with target cells is not strong, we have developed another efficient screening method to identify virus entry factors (hereafter referred to as 2^nd^ generation panning for virus entry factor identification or simply 2^nd^ generation panning)^32,33^. In 2^nd^ generation panning, same as in 1^st^ generation panning, cellular cDNA libraries are stably expressed in non-adherent cell lines by using a retroviral vector, but two additional non- or low-cytotoxic retroviral/lentiviral vectors which bear intended viral envelope proteins and whose genome encode either a membrane spanning protein or a fluorescence protein as a reporter are used for screening. Upon inoculation of library-expressing cells with a first screening vector encoding a membrane spanning protein, target cells would be specifically infected with the vector, express the reporter stably on cell surface, and form colonies on anti-reporter antibody-coated dishes during cell culture. Because infrequent infection of non-target cells with the screening vectors would occur and result in colony formation as authentic target cells, resultant colonies are superinfected with a second screening vector whose genome encode a fluorescence protein to eliminate false positive. Fluorescent colonies are found out under a fluorescence microscope and inserted cDNA recovered by PCR like with 1^st^ generation panning. The ability to produce moderate to high titres of retroviral/lentiviral vectors bearing intended viral envelope proteins (as a guide, ≥10^5^ infectious units [IU]/mL) is a perquisite for 2^nd^ generation panning. However, we have experienced cases in which some viruses (*e.g*., West Nile virus) did not meet the prerequisites for either method and for which virus entry factors could not be identified by our methods (data not shown).

In the current taxonomy SFTS virus is classified as follows: species, *Huaiyangshan banyangvirus*; genus, *Banyangvirus*; family, *Phenuiviridae*, and order, *Bunyavirales* (https://talk.ictvonline.org/taxonomy/). In members of this genus, non-evident cytopathic effects are characteristically observed in *in vitro* short cell culture^15,34–36^. The genome of the genus members is composed of three negative sense RNAs of large (L), middle (M), and small (S) segments, which encode viral proteins (RNA-dependent RNA polymerase, glycoprotein [GP], and nuclear and non-structural proteins, respectively). The rescue of SFTS virus with or without mutations from cDNA (reverse genetics) has been reported^37^; in that study, five plasmids expressing three anti-genome RNAs and two viral proteins (RNA-dependent RNA polymerase and nuclear protein) were used. As an application of the reverse genetics, a virus-like particle (VLP) assay was recently reported to assess the reassortment potential of SFTS virus with its related viruses Heartland virus (a member of the same genus) and Uukuniemi virus (a member of the genus *Phlebovirus* of the same family)^38^. Crimean-Congo haemorrhagic fever (CCHF) virus, a member of the family *Nairoviridae* of the same order, has similar characteristics to SFTS virus with regard to cytopathic effectivity, genome composition, transmission modes, and disease manifestations^39–41^.

The methods used for identification of SFTS virus entry factors to date are classified into categories I (C-type lectins^29,30^), II with loss-of-function criteria (glucosylceramide and SNX11^32,33^), and III (NMMHC-IIA^31^) described above. However, there are no reports on the application of category II methods with gain-of-function criteria in the identification of SFTS virus entry factors. In this report, we show the success of cellular cDNA library screening to identify SFTS virus entry factors with a novel method, which is combination of our 2^nd^ generation panning^32,33^ and the reverse genetics for SFTS virus^37,38^ and is the first category II method with gain-of-function criteria applied for SFTS virus. Its application in the identification of previously unidentified SFTS virus entry factor(s), as well as entry factor(s) for viruses related to SFTS virus will be discussed.

## Results

### First and second generation panning for the identification of SFTS virus entry factors

**Figure 1:**
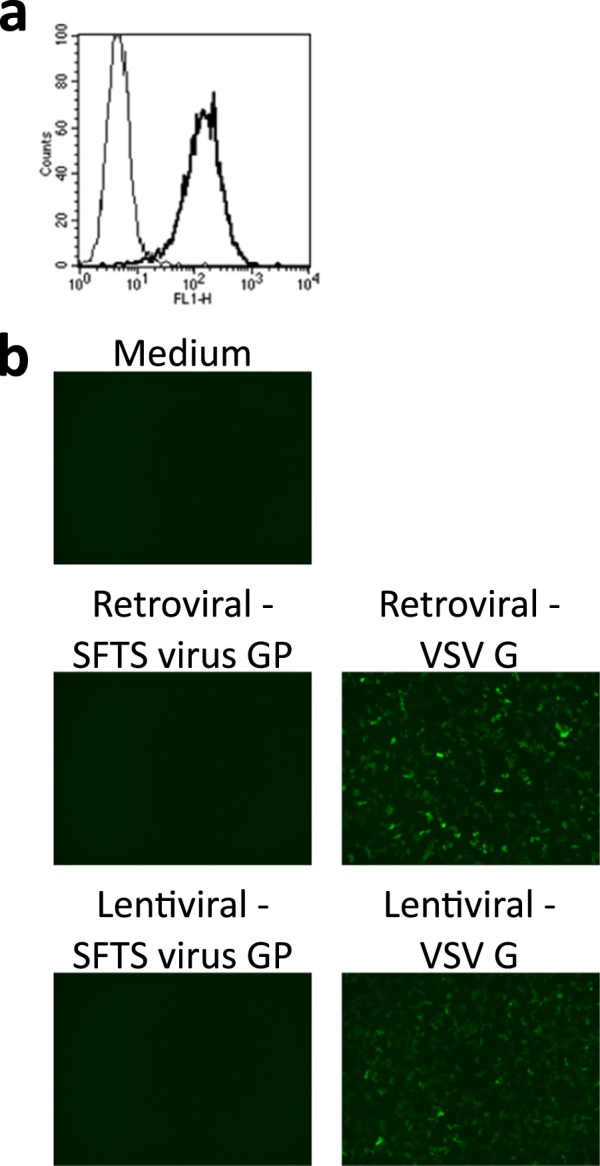
First and second generation panning for the identification of virus entry factors (**a**) Vero cells were mixed with medium (thin line) or SFTS virus (bold line) on ice and SFTS virus on the cell surface was detected by flow cytometry. (**b**) The infectivity of retroviral and lentiviral vectors prepared with SFTS virus glycoprotein (GP) or vesicular stomatitis virus G (VSV G), whose reporter was enhanced green fluorescence protein or Venus, was examined in Vero cells by fluorescence microscopy.

We first tried to identify SFTS virus entry factor(s) with one of our previously reported methods (1^st^ generation panning)^28–30^. In flow cytometry, the binding of SFTS virus particles to Vero cells, an SFTS virus-highly susceptible cell line^8,12,14^, was observed (Fig. 1a). However, Petri dishes pre-coated with SFTS virus particles were not able to trap Vero cells (data not shown). These findings indicated that the interaction observed between SFTS virus particles and entry factor(s) on Vero cells was not strong enough to trap Vero cells on the panning dishes. Thus, 1^st^ generation panning could not be applied in the identification of SFTS virus entry factors.

Next, we examined the usability of 2^nd^ generation panning^32,33^ to identify SFTS virus entry factor(s). Retroviral and lentiviral vectors were prepared with an SFTS virus GP-expression plasmid, as described in the Methods. Vero cells were inoculated with media containing the vectors at a dilution of 1:5, but no apparent reporter expression (enhanced green fluorescence protein or Venus^42^) was observed under a fluorescence microscope (Fig. 1b). In contrast, the usage of a vesicular stomatitis virus G (VSV G)-expression plasmid for the preparation of retroviral/lentiviral vectors resulted in moderate reporter expression (Fig. 1b). The retroviral/lentiviral vector titres determined by flow cytometry (Supplementary Fig. S1) were as follows: 2.5 ×10^2^ IU/mL (retroviral – SFTS virus GP), 5.0 × 10^2^ IU/mL (lentiviral – SFTS virus GP), 2.9 × 10^5^ IU/mL (retroviral – VSV G), and 6.7 × 10^5^ IU/mL (lentiviral – VSV G) (detection limit of the assay: 2.5 × 10^2^ IU/mL). These indicated that the titres of retroviral/lentiviral vectors conferred by SFTS virus GP were considerably low, meaning that it was difficult to apply 2^nd^ generation panning for the identification of SFTS virus entry factors.

Thus, these panning-based screening methods would need to be improved or other methods would need to be used to identify SFTS virus entry factor(s).

### Characterisation of SFTS virus-based infectious virus-like particle (VLP) carrying Venus reporter gene

**Figure 2:**
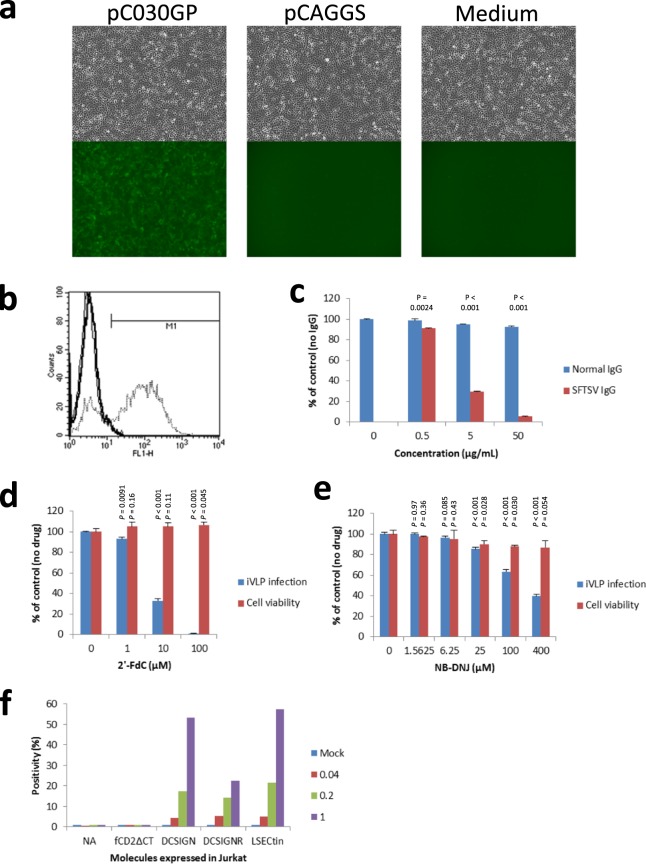
Characterisation of SFTS virus-based infectious virus-like particle (iVLP) carrying Venus reporter gene (**a**,**b**) Vero cells were inoculated with iVLP prepared with (pC030GP, dotted line) or without (pCAGGS, bold line) SFTS virus GP-expression or inoculated with medium (Medium, thin line) and observed under a fluorescence microscope (**a**) or analysed by flowcytometry (**b**). (**c**) Inoculation with iVLP was performed with pretreatment with normal human IgG or SFTS virus-neutralising monkey IgG. iVLP infection was quantified as fluorescence positivity by flow cytometry. The experiment was performed in triplicate and the infection relative to control (no IgG) is shown in means (%) + standard deviations (s.d.). Statistical comparison was performed between normal IgG and SFTSV IgG (Welch’s *t*-test, two-tailed, n = 3). (**d**,**e**) Inoculation with iVLP was performed in the presence of a nucleoside analogue (2′-deoxy-2′-fluorocytidine [2′-FdC]) (**d**) or a glucosylceramide synthase inhibitor N-butyldeoxynojirimycin. (**e**) The infection was quantified and analysed as in. (**c**) Cell viability was also measured in triplicate, and viability relative to control (no drug) is shown in means (%) + s.d. Statistical comparison was performed between control and drug treatment (Welch’s *t*-test, two-tailed, n = 3). (**f**) Jurkat cells or Jurkat cells expressing control molecule (fCD2ΔCT) or C-type lectins were inoculated with iVLP(Venus) at the indicated MOIs and analysed by flow cytometry. Data shown are means of duplicate experiments. NA, not available.

A VLP assay using components of SFTS virus was recently reported. In this assay, luciferase was used as a reporter to quantify VLP infection^38^. In the assay, luciferase activity mirrors both the entry level of VLP and the replication and transcription levels of the luciferase gene-coding viral segment in VLP-inoculated cell populations. To develop an assay to identify SFTS virus entry factors, in which the identification or isolation of reporter-expressing cells among the VLP-inoculated cell population would be required, we used a fluorescence protein, Venus^42^, as a reporter. SFTS virus-based infectious VLP (iVLP) was prepared as described in the Methods and used to inoculate Vero cells. On the day after inoculation, inoculated cells showed fluorescence under a fluorescence microscope, as demonstrated in Fig. 2a (left). Additionally, the observation of the morphology of the inoculated cells revealed were no evident cytopathic effects (*e.g*., cell-rounding) in the inoculated cells. In contrast, when inoculated with iVLP, which was prepared with empty pCAGGS instead of pC030GP for the envelope protein expression (Fig. 2a, centre) or inoculated with medium (Fig. 2a, right), Vero cells showed no fluorescence. Flow cytometry confirmed expression of a fluorescence protein in cells inoculated with iVLP that was prepared with pC030GP (Fig. 2b). The iVLP titre determined with Vero cells was 6.0 × 10^5^ IU/mL. The results strongly suggested that the observed fluorescence was derived from a reporter of iVLP (Venus), that its expression was SFTS virus GP-dependent, and that the identification of iVLP-infected cells would be possible.

Furthermore, to examine the dependency of the observed reporter expression on SFTS virus components, inoculation with iVLP was performed with antibody pretreatment or in the presence of inhibitory compounds. iVLP was premixed with normal human IgG or SFTS virus-neutralising monkey IgG then inoculated onto Vero cells and cultured overnight. As shown in Fig. 2c, SFTS virus-neutralising IgG, but not normal human IgG, reduced the reporter expression upon iVLP inoculation in a dose-dependent manner. The neutralising IgG within similar ranges showed the inhibitory effects also on authentic SFTS virus infection (Supplementary Fig. S2). A nucleoside analogue 2’-deoxy-2’-fluorocytidine (2’-FdC) has been reported to have strong inhibition activity against SFTS virus infection; the underlying mechanism of this is probably the inhibition of transcription/replication of the SFTS virus genome^43^. Vero cells were inoculated with iVLP in the presence of 2’-FdC. As shown in Fig. 2d, 2’-FdC reduced the reporter expression upon iVLP inoculation in a dose-dependent manner without cytotoxicity. An inhibitor of glucosylceramide synthase, N-butyldeoxynojirimycin, was reported to reduce intracellular trafficking and/or penetrating process of SFTS virus infection^16^. The inhibitor, as shown in Fig. 2e, reduced the reporter expression upon iVLP inoculation in dose-dependent manners, although showed slight cytotoxicity at high doses (*e.g*. 25 and 100 μM). We previously reported that SFTS virus proliferation in Jurkat cells is enhanced by expression of C-type lectins (*i.e*., dendritic cell-specific ICAM-3 grabbing non-integrin [DC-SIGN], DC-SIGN-related [DC-SIGNR], and liver and lymph node sinusoidal endotherial cell C-type lectin [LSECtin]) in the cells^14^. Therefore, we compared Jurkat cell susceptibility to iVLP with or without C-type lectin expression. Expression of each C-type lectin in Jurkat cells analysed by flow cytometry is shown in Supplementary Fig. S3. As shown in Fig. 2f, a control molecule (fCD2ΔCT) did not enhance susceptibility of Jurkat cells to iVLP infection, however, in contrast, any of C-type lectins examined did.

Thus, the reporter expression in iVLP-inoculated cells occurred through the same mechanisms as cell entry to genome transcription/replication steps during authentic SFTS virus infection.

### Usage of iVLP in a screening model to identify virus entry factors

The absence of evident cytopathic effects in iVLP-infected cells (Fig. 2a) was reminiscent of retroviral/lentiviral vectors that are well known to express intended genes stably with quite low cytotoxicity. With the combination of panning and the retroviral/lentiviral vectors, we developed an efficient method for identifying the entry factors of Ebola virus and Lassa virus, in which entry factor-expressing cells were isolated from other cells by panning and cultured for approximately one week for colony-formation in order to reduce false-positive selection (2^nd^ generation panning)^32,33^.

**Figure 3:**
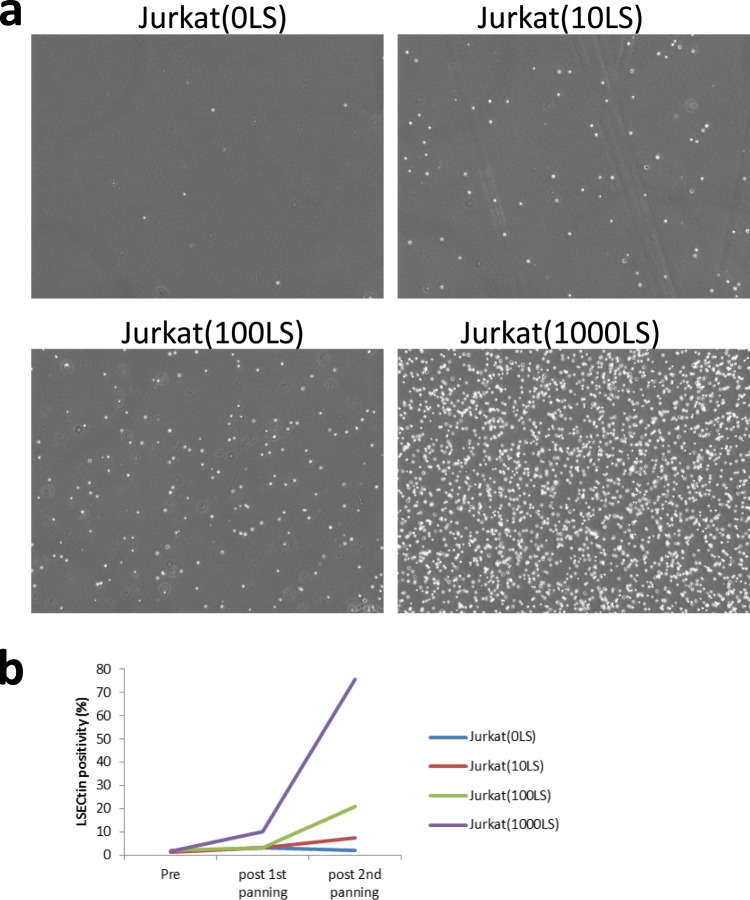
A screening model to identify SFTS virus entry factors. (**a**) Jurkat cells (1.6 × 10^6^) mixed with 0, 10, 100, and 1,000 Jurkat cells expressing LSECtin (Jurkat[0LS], Jurkat[10LS], Jurkat[100LS], and Jurkat[1000LS], respectively) were inoculated with an infectious virus-like particle carrying fCD2ΔCT reporter gene (iVLP[fCD2ΔCT]) at a multiplicity of infection of 1 and subjected to panning. Cells that were trapped but later detached were harvested, cultured, then re-inoculated with iVLP(fCD2ΔCT), followed by panning again. Trapped cells in the 2^nd^-round of panning, are shown in (**a**). (**b**) Positivity (%) by flow cytometry with anti-LSECtin antibody was measured for each cell mixture before panning (pre) or after the 1^st^ (post 1^st^ panning) or 2^nd^ (post 2^nd^ panning) panning.

We investigated whether SFTSV iVLP could work like retroviral/lentiviral vectors in the identification of virus entry factors. As a model experiment, 1.6 × 10^6^ Jurkat cells were mixed with 0, 10, 100, and 1,000 Jurkat cells expressing LSECtin (Jurkat[0LS], Jurkat[10LS], Jurkat[100LS], and Jurkat[1000LS], respectively), which is a C-type lectin that confers SFTS virus-susceptibility to Jurkat cells^30^. The 4 cell mixtures were separately inoculated with iVLP carrying fCD2ΔCT reporter gene (iVLP[fCD2ΔCT]) at a multiplicity of infection (MOI) of 1 and on the following day subjected to panning with anti-fCD2 antibody. Trapped cells started to divide in the subsequent cell culture; however, 1 week later they detached from the antibody-coated panning dishes (data not shown). The detached cells were harvested, cultured for one additional week, then re-inoculated with iVLP(fCD2ΔCT). Panning was then performed again. In this 2^nd^-round of panning, as shown in Fig. 3a, the numbers of trapped cells increased in ascending order as follows: Jurkat(0LS), Jurkat(10LS), Jurkat(100LS), and Jurkat(1000LS). Accumulation of LSECtin-expressing cells by repetition of iVLP infection and panning was confirmed by flow cytometry with anti-LSECtin antibody (Fig. 3b). The results indicated that iVLP-infected cells were able to continue cell division but that the reporter expression was reduced as cell division proceeded. Thus, SFTS virus-based iVLP could replace the retroviral/lentiviral vectors in 2^nd^ generation panning for the identification of virus entry factors^32,33^; however, colonies should be picked up within 1 week after panning.

### Human liver cDNA library screening using iVLPs

**Figure 4:**
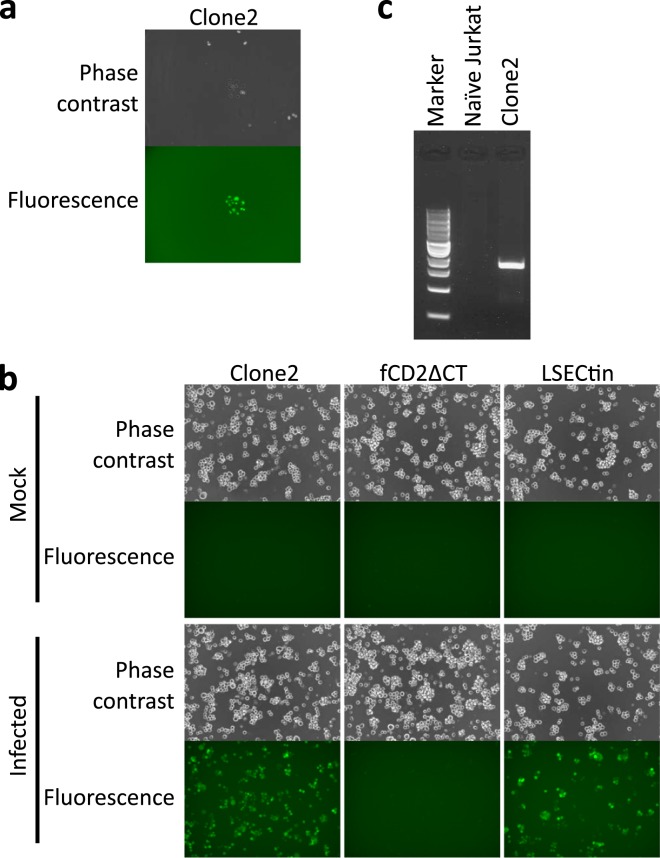
Human liver cDNA library screening using infectious virus-like particle (iVLP). (**a**) In screening of the human liver cDNA library to identify SFTS virus entry factors, six Jurkat colonies (Clones1–6), which consisted of Venus-expressing cells, were found on a panning dish and one of them (Clone2) is shown. (**b**) Propagated Clone2, control molecule (fCD2ΔCT)-expressing Jurkat cells and LSECtin-expressing Jurkat cells were examined to check their susceptibility to iVLP(Venus). (**c**) The genomes of naïve and Clone2 were subjected to polymerase chain reaction with a pMX primer pair. A 1-kb DNA ladder was used as a marker.

Based on the results of the model experiment described above, we performed screening of a human liver cDNA library to identify SFTS virus entry factor(s). Jurkat cells (1.0 × 10^7^) expressing a human liver cDNA library were inoculated with iVLP(fCD2ΔCT), then subjected to panning on the following day. On the third day after panning, the Jurkat colonies that had formed on the panning dish were inoculated with iVLP(Venus). The next day, under a fluorescence microscope, six Jurkat colonies (Clone1–6) in which most cells expressed Venus were identified (Fig. 4a, Supplementary Fig. S4) and picked up separately. Among the six colonies, unfortunately, only Clone2 could propagate. The propagated Clone2, control molecule (fCD2ΔCT)-expressing Jurkat cells and LSECtin-expressing Jurkat cells were examined to evaluate their susceptibility to iVLP(Venus) infection. As shown in Fig. 4b, Clone2 and LSECtin-expressing cells, but not control molecule-expressing Jurkat cells, showed iVLP(Venus)-susceptibility. PCR with a pMX primer pair amplified an approximately 2 kb fragment from the Clone2 cellular genome (Fig. 4c) the sequence of which was determined to be a variant of DC-SIGNR, which had 4 tandem-repeats of 23 amino acids in the neck region^44,45^.

We repeated library screening on a larger scale (1.0 × 10^8^ Jurkat cells expressing human liver cDNA library) and obtained 49 colonies after iVLP(Venus) inoculation. To expand the colonies, 10 naïve Jurkat cells were added to each colony culture. All mixtures grew but only 24 showed susceptibility to iVLP(Venus) infection in a susceptibility-confirmation test (data not shown). Among the 24 clones, 16 clones were revealed to have variants of DC-SIGNR cDNA originating from the liver cDNA library in their genome: 10 and 6 clones with 4 and 7 tandem-repeats, respectively, in the neck region of DC-SIGNR. With the genome of the remaining 8 clones, however, no cDNA originating from the liver could be rescued.

### Two DC-SIGNR variants identified and SFTS virus infection

**Figure 5:**
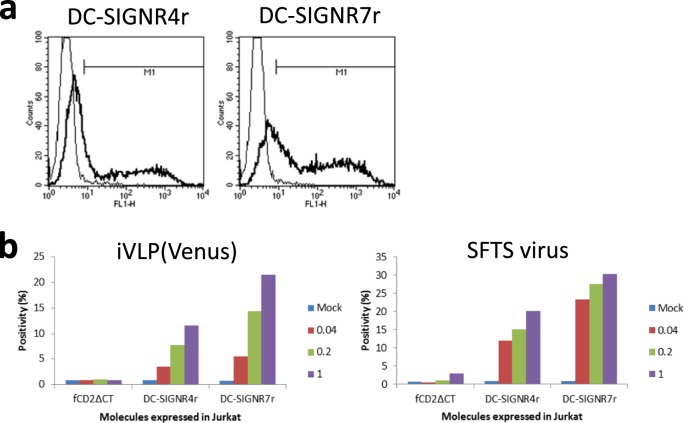
Two DC-SIGNR variants identified and SFTS virus infection. (**a**) DC-SIGNR4r (left) and DC-SIGNR7r (right) were expressed in Jurkat cells by lentivirus vectors and their expressions were analysed by flow cytometry with control IgG (thin line) and anti-DC-SIGNR IgG (bold line). (**b**) Jurkat cells expressing a control molecule (fCD2ΔCT), DC-SIGNR4r, or DC-SIGNR7r were inoculated with iVLP(Venus) (left) or authentic SFTS virus (right) at the indicated MOIs and the expression of a reporter (Venus) or a viral protein (NP) in the cells was examined by flow cytometry. Data shown are means of duplicate experiments.

A most common form of DC-SIGNR has 7 tandem-repeats in the neck region^44–46^, and so did DC-SIGNR used in the characterisation of iVLP in this report (*e.g*. Fig. 2f). The two DC-SIGNR variants with 4 (DC-SIGNR4r) and 7 (DC-SIGNR7r) tandem-repeats in the neck region were expressed in Jurkat cells by using lentivirus vectors and susceptibilities of the cells to infection of iVLP(Venus) and SFTS virus were examined. Expression of each variant was confirmed by flow cytometry, with DC-SIGNR7r showing higher positivity (65%) than DC-SIGNR4r (35%, Fig. 5a). When inoculated, MOI-dependent expression of a reporter (Venus by iVLP[Venus]) or a viral protein (nuclear protein by SFTS virus) were observed within Jurkat cells expressing either of the two variants (Fig. 5b). The higher positivity of the reporter expression in DC-SIGNR7r-expressing cells than in DC-SIGNR4r-expressing cells (Fig. 5b) correlated with the variant positivity (Fig. 5a). These results indicated that cellular molecules identified by a method reported here indeed involved SFTS virus infection and suggested that no apparent difference existed between the two variants with regard to enhancement of SFTS virus infection.

## Discussion

We tried but failed to identify SFTS virus entry factors using our 1^st^ and 2^nd^ generation panning methods, which were previously used for other viruses^28–30,32,33^. We focused on the established reverse genetics of the SFTS virus for viral genome manipulation^37,38^ and on a characteristic of SFTS virus that the virus shows no or weak cytotoxicity in infected cells^15,34^ and investigated whether SFTS virus-based vectors (iVLPs), like retroviral/lentiviral vectors, could be useful for identifying SFTS virus entry factors. Although the reporter expression by iVLP did not continue for more than one week, we screened a liver cDNA library with iVLPs and succeeded in identifying a C-type lectin, DC-SIGNR, which has been reported as an entry-enhancing factor^13,14^. Thus, even though the envelope protein of the virus did not show strong binding to the cell surface and conferred no or low retroviral/lentiviral titres, we showed that efficient gain-of-function screening for virus entry factor(s) could be performed using a VLP based on the virus itself (hereafter referred to as 3^rd^ generation panning for virus entry factor identification or simply 3^rd^ generation panning). To our knowledge, this is the first report on gain-of-function-based entry factor screening methods for SFTS virus. A prerequisite for 3^rd^ generation panning is that the VLP does not significantly inhibit cell growth upon infection (for at least a few days), because the growth of cells, which obtain susceptibility to the intended viruses through the induction of cDNA clones and become infected with iVLP, is required for efficient selection^32,33^. From this viewpoint, it is unlikely that a vesicular stomatitis virus (VSV)-based pseudotyping system would work for such gain-of-function screening due to the high cytotoxicity of VSV replication, whereas envelope proteins of many viruses, including SFTS virus, confer infectivity to VSV-pseudotypes^13,14^. Another prerequisite is that moderate titres of VLP can be obtained—as a guide, VLP titres of ≥10^5^ IU/mL without concentration are desired, because in screening approximately 10^7^ library-expressing cells are generally needed in order to be infected at an MOI of 1^28–30,32,33^. Usability of SFTS virus-based iVLP as a backbone is now under investigation to analyse entry mechanisms and/or viral particle-formation mechanisms and/or identify entry factors for other envelope viruses. Although we used SFTS virus as a backbone of the VLP for virus entry factor identification, the usage of VLPs based on other viruses (*e.g*., Guertu virus, Heartland virus and Uukuniemi virus, members of the same genus or family as SFTS virus) would be acceptable if the prerequisites are fulfilled. In particular, CCHF virus (like SFTS virus) shows no evident cytopathic effects^39–41^ and a VLP assay based on reverse genetics is available^47^. There is limited information on the entry mechanism of CCHF virus^48,49^ or the pathogenesis of CCHF^39^. We observed that glycoprotein of CCHF virus conferred relatively low retroviral/lentiviral vectors titres (data not shown). In addition, the disease manifestations in CCHF and SFTS patients display a large degree of overlap^8,39^. Thus, identification of CCHF virus entry factors using CCHF virus-based (or other virus-based) VLP might be feasible and may help to understand the pathogeneses of CCHF and SFTS.

The DC-SIGNR molecule has considerable polymorphism, especially within its neck region, which includes 3 to 9 highly conserved repeats containing 23 amino acids^44–46^ and the polymorphism may affect host susceptibility to infection with some pathogens^46,50^. In the present study with a liver cDNA library, two variants of DC-SIGNR with 4 and 7 repeats in the neck regions were identified as SFTS virus entry factors, suggesting that polymorphism of DC-SIGNR may not be important in DC-SIGNR-mediated SFTS virus infection. Because the cDNA library used was prepared from a male, the two identified variants were due to the donor’s DC-SIGNR alleles.

It is presently unclear whether C-type lectins, including DC-SIGNR, can work as receptors for SFTS virus or as enhancers that facilitate receptor-mediated SFTS virus infection. A study with SFTS virus-related Uukuniemi virus showed the differential use of C-type lectins (DC-SIGN and DC-SIGNR) for its endocytosis^51^. Nevertheless, because SFTS virus replicates efficiently in cell lines that do not express C-type lectins (*e.g*., Vero and Huh cells) *in vitro*^8,12–14^, other yet unidentified cellular factors are likely to be involved in SFTS virus cell entry. There are several possible reasons why no molecule rather than DC-SIGNR was identified in the present study: *e.g*., cDNA clones encoding other entry factors might be not included or truncated in the cDNA library used due to the length (long cDNAs tend to be missed or damaged during cDNA library preparation and/or retrovirus vector packaging) or there might be a viral entry route(s) for which more than one cellular molecules were necessary but lacked in cells used for library assessment (simultaneous identification of two or more molecules involving a single entry route is quite difficult in gain-of-function screening). Thus, Vero and Huh cells will be usable as sources of cDNA libraries. In addition, Raji and Molt-4 cells, which are highly resistant to SFTS virus infection^13,14^, may be usable as target cells to assess cDNA libraries. The use of different combinations of cDNA library origins/target cells rather than liver/Jurkat cells, which was used in this report, might lead to the identification of unidentified cellular factors involved in SFTS virus cell entry and may eventually lead to a better understanding of the viral tropism and the pathogenesis of SFTS.

In the small scale-library screening, 5 of the 6 cellular clones that were picked up did not grow in the subsequent cell culture. In the large scale-library screening, the addition of naïve cells supported the growth of 24 of 49 iVLP-infected cell clones; however, in the remaining 25 cell cultures it is likely that only naïve Jurkat cells grew. Thus, infection with SFTS virus-based iVLP affected cell growth somewhat. The usage of prolactin^52^ or non-dividing feeder cells or the usage of inhibitors of viral genome replication/transcription such as 2’-FdC^43^ to support the growth of the cells that are picked up might improve screening efficiency.

Two recombinant SFTS viruses, similar to iVLP, which express reporters in inoculated cells, have been reported. One is replication-competent SFTS virus whose genome encodes non-structural protein-reporter fusion protein or lacks non-structural protein gene but encodes a reporter^53^. Use of this virus for entry factor screening has never been reported but would probably result in low efficiency due to target cell damage and/or high background caused by its replication competency. The other is replication-incompetent SFTS virus whose genome encodes only reporters^38^. Because sufficient reporter expression upon the viral infection needs *in-trans* expression of viral RNA-dependent RNA polymerase and nuclear protein in inoculated cells^38^, entry factor screening with this virus would be considerably complicated and the range of target cells usable for screening would be narrow. Because the genome of iVLP reported here encodes all viral proteins except GP (see Methods), iVLP is expected to be replication incompetent but does not require *in-trans* expression of viral proteins for detection of reporter in inoculated cells. Therefore, we believe that iVLP is the best tool for conducting gain-of-function screening to identify SFTS virus entry factors.

We reported a screening method to identify virus entry factors; however, theoretically, the method could be applicable to the identification of host factors involved in replication/transcription of the viral genome. The partial inhibition of the replication/transcription of the SFTS viral genome via innate immunity was observed in rodent cells^54^; thus, truncated forms affecting the inhibition of replication/transcription might be identified using this screening method. In fact, we previously discovered a novel role of ribosomal protein S6 kinase alpha 3 (RSK2) in innate immune responses to influenza virus infection through identification of truncated RSK2 which dominates full length RSK2 and eventually increases reporter expression driven by influenza virus genome replication/transcription^55^. The identification of factors involved in the replication/transcription of the SFTS viral genome will advance the understanding of cellular anti-SFTS virus mechanisms.

In the present study we reported the successful identification of SFTS virus entry factors by establishing a novel, efficient virus entry factor screening method using SFTS virus-based infectious VLP. This method may be a key to understanding not only the viral tropism and pathogenesis of SFTS but also that of other viruses.

## Methods

### Cells

Vero cells (ATCC CCL-81) were cultured in DMEM (Sigma) supplemented with 5% heat-inactivated foetal calf serum (FCS) and antibiotics (Gibco, Pen Strep). Upon inoculation, Vero cells were maintained in DMEM with 2% FCS (DMEM-2FCS) and antibiotics. Jurkat cells (Clone E6-1, ATCC TIB-152) were cultured and maintained in RPMI1640 (Sigma) supplemented with 10% FCS and antibiotics. BSR-T7/5 cells^56^ were kindly provided by Dr. Karl-Klaus Conzelmann and cultured in DMEM supplemented with 10% FCS, 10% tryptose phosphate broth, antibiotics, and 1 mg/mL of G418 (Sigma, in every other passage). Jurkat cells expressing LSECtin or a control molecule were prepared using a lentiviral vector as described previously^32^.

### Reagents

Anti-feline CD2 monoclonal antibody SKR2 and anti-SFTS virus nuclear protein monoclonal antibody 9D3 were reported previously^57,58^. Purified SFTS virus-neutralising antibody was prepared from sera of monkeys that were inoculated with SFTS virus using Protein G Sepharose 4 Fast Flow (GE Healthcare) (Shimojima *et al*., manuscript in preparation). Mouse anti-SFTS virus GP monoclonal antibodies (Immune Technology Corp., clones 62D6 and 21D2), mouse IgG1 isotype control (R&D Systems), mouse anti-DC-SIGN/DC-SIGNR (clone DC28, R&D Systems), mouse anti-LSECtin monoclonal antibody (clone SOTO-1, Santa Cruz Biotechnology), normal human IgG (Wako Pure Chemical Industries, Ltd.), goat anti-mouse IgG (H + L) Alexa Fluor488 (Thermo Fisher Scientific), 2′-deoxy-2′-fluorocytidine hydrate (Tokyo Chemical Industry Co., Ltd.), N-butyldeoxynojirimycin (Cayman Chemical), WST-1 (Sigma-Aldrich) and 1 kb DNA Ladder (New England Biolabs Japan) were purchased.

### Flow cytometry for SFTS virus binding

Adherent Vero cells were detached and suspended as described previously^34^. Suspended cells were mixed with DMEM-2FCS containing no virus or SFTS virus SPL030 strain^12^ at a ratio of 100 times the 50% tissue culture infectious dose/cell on ice for 1 h with occasional mixing. After washing with DMEM-2FCS, cells were fixed in 10% formalin for 1 h at room temperature. The incubation of fixed cells with primary anti-SFTS virus monoclonal antibody and secondary goat anti-mouse IgG (H + L) Alexa Fluor488 was performed as described previously^32^. A FACSCalibur system and Cell QuestPro software program were used to analyse labelled cells.

### Flow cytometry with intracellular staining

To detect intracellular viral proteins, formalin-fixed cells were treated and stained with antibodies with BD Perm/Wash™ Buffer (Thermo Fisher Scientific) according to the manufacture’s protocol. Labelled cells were analysed as described above.

### Coating with SFTS virus and panning

Petri dishes were first coated with anti-mouse IgG antibody followed by anti-SFTS virus antibody and SFTS virus, as described previously^28^. Vero cells were suspended in cold DMEM-2FCS (described in the Flow cytometry for SFTS virus binding section) and put onto SFTS virus-coated dishes. The dishes with Vero cells were incubated in a refrigerator for 30 min and observed under a microscope to assess the adhesion of the Vero cells to the virus-coated dishes.

### Preparation of retroviral/lentiviral vectors

Murine leukaemia virus (MLV)-based retroviral and human immunodeficiency virus type 1-based lentiviral vectors were prepared by transfection of plasmids as described previously^32,33^. GP cDNA cloned in pCAGGS (pC030GP)^34^ was used to express SFTS virus GP. Titres of retroviral/lentiviral vectors were calculated from both the positivity of Vero cells inoculated (2 days post-inoculation) in flow cytometry and the numbers of Vero cells used.

### Expression of cDNA library

The human liver cDNA library in the pFB plasmid was purchased from Stratagene (La Jolla, CA). MLV-based retroviral vectors carrying the library were prepared as described previously^32^ and were then used to infect Jurkat cells at an MOI of 0.2.

### Infectious virus-like particles (iVLPs) carrying reporter gene

Plasmids for SFTS virus reverse genetics (HB29 strain) were kindly provided by Dr. Benjamin Brennan^37^. Inserts of pTVT7-HB29L, pTVT7-HB29ppM, and pTVT7-HB29S were replaced with the L, M, and S segment cDNAs of the SFTS virus SPL030 strain genome^12^ (pTV7OriL, pTV7OriM, pTV7OriS). The open reading frame of GP in pTV7OriM was replaced with the cDNA of a fluorescence protein Venus^42^ or feline CD2 lacking cytoplasmic tail^57^ (pTV7MVenus and pTV7MfCD2ΔCT). To produce iVLPs, plasmids pTM1-HB29ppL, pTM1-HB29N, pTV7OriL, pTV7OriS, pC030GP, and pTV7MVenus or pTV7MfCD2ΔCT were co-transfected to BSR-T7/5 cells using *Trans*IT-LT1 reagent (Takara). The supernatant of the transfected cells was harvested at 4 days post-transfection and used directly or after concentration with Amicon Ultra 100 K MWCO (Merck).

To titrate iVLPs, serially diluted iVLP(Venus) were inoculated onto Vero cells and the reporter expression was analysed by flow cytometry. iVLP(fCD2ΔCT) was prepared in parallel with iVLP(Venus) and the titre of iVLP(fCD2ΔCT) was considered to be the same as that of iVLP(Venus).

### Library screening

cDNA library-expressing Jurkat cells were inoculated with iVLP(fCD2ΔCT) at an MOI of 1. The next day, panning was performed with anti-fCD2 antibody, as described previously^59^. Panning dishes were cultured with RPMI1640 medium containing 10% FCS and antibiotics for 3 to 4 days. Colony-forming cells were further inoculated with iVLP(Venus). The next day, colony-forming Venus-positive cells were picked up for propagation followed by genome extraction using DNAzol (Molecular Research Center). To recover integrated cDNAs, the extracted genome was subjected to PCR with a pMX primer pair (pMX5F: 5′-GGTGGACCATCCTCTAGACT-3′ and pMX3R: 5′- CCCTTTTTCTGGAGACTAAAT-3′)^59^ and PrimeSTAR Max DNA Polymerase (Takara) under the following conditions: 98 °C for 2 min followed by 35 cycles of 98 °C for 10 s, 55 °C for 5 s, and 72 °C for 30 s. The sequences of the PCR products were determined by Sanger’s method with the pMX primer.

### Equipment and settings for imaging

Images of cells were obtained as TIFF 8-bit by using a fluorescence microscope BZ-X710 and BZ-X Viewer software (KEYENCE). Fluorescence was detected by the single colour setting (488 nm). Images of DNA gel electrophoresis were obtained by using FAS-III (TOYOBO) with GelRed nucleic acid gel stain reagent (Biotium).

### Cell viability

Cell viability was examined by using WST-1 reagent (Sigma-Aldrich) according to the manufacturer’s protocol.

### Statistical analyses

Welch’s *t*-test was used to determine the significance of the difference between the means of two groups. A value of *P* < 0.05 was considered to indicate statistical significance.

## Supplementary information

Supplementary information

## Data Availability

No datasets were generated or analysed during the current study.
