# Supplementary material for: Efficient functional screening of a cellular cDNA library to identify severe fever with thrombocytopenia syndrome virus entry factors
Source: Sci Rep. 2020 Apr 7;10:5996. doi: 10.1038/s41598-020-62876-1 (PMC7138800; doi:10.1038/s41598-020-62876-1)
Supplement: Supplementary file 1 — Supplementary information [file 41598_2020_62876_MOESM1_ESM.pdf]

# Efficient functional screening of a cellular cDNA library to identify severe fever with thrombocytopenia syndrome virus entry factors

Shimojima, M., Sugimoto, S., Taniguchi, S., Yoshikawa, T., Kurosu, T., Saijo, M.

Supplementary Figs. S1 to S4

**a** Retroviral **b** Lentiviral

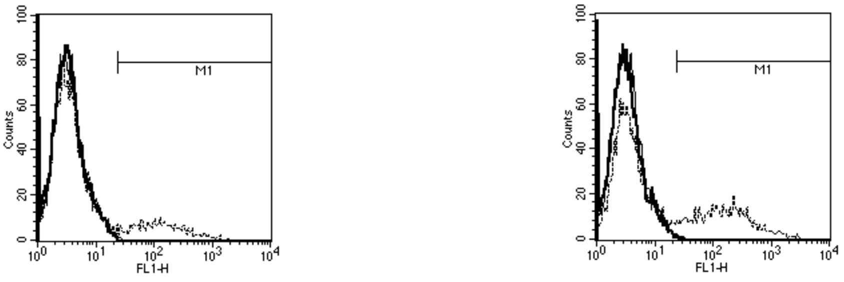


**c**

| Inoculum | Positivity by FCM (%) |
|---|---|
| Retroviral – SFTS virus GP 1:5 | 0.00 |
| Retroviral –VSV G 1:5 | 11.41 |
| Lentiviral – SFTS virus GP 1:5 | 0.02 |
| Lentiviral – VSV G 1:5 | 26.91 |

**Supplementary Fig. S1: Infectivity of retroviral and lentiviral vectors in Vero cells**
Vero cells were inoculated with retroviral (a)/lentiviral (b) vectors prepared with SFTS virus GP (bold line) or with VSV G (dotted line) at 1:5 dilution and reporter expression was measured by flowcytometry. Vero cells were also inoculated with medium (thin line) as mock. (c) Positivity (%) of inoculated cells by flowcytometry are shown.

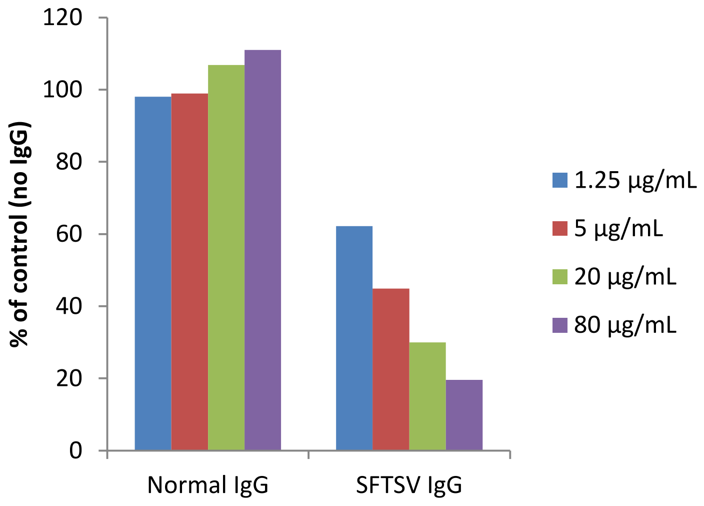


**Supplementary Fig. S2: Effects of normal human IgG (Normal IgG) and monkey neutralising IgG (SFTSV IgG) on infection of authentic SFTS virus**

Neutralising effects of IgG on SFTS virus infection were measured with a method reported by Taniguchi et al. (J Virol Methods. 2017;244:4-10). Means of duplicate experiments are shown.

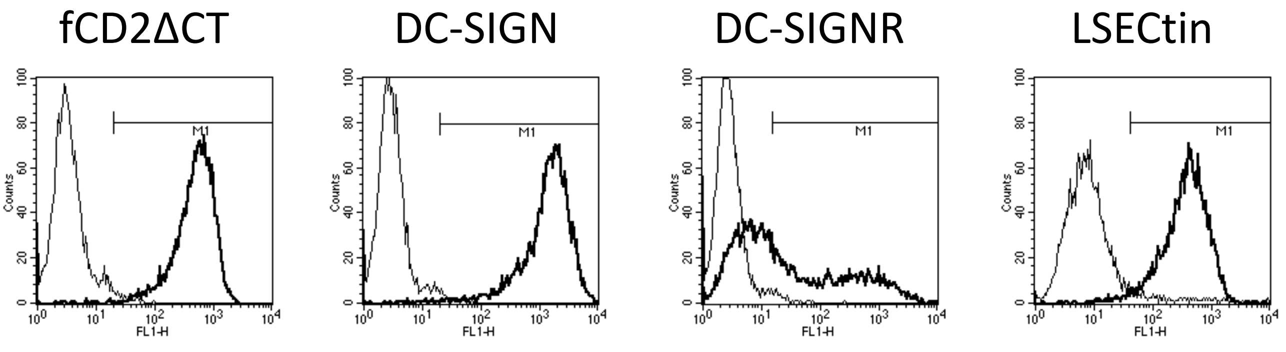


**Supplementary Fig. S3: Expression of C-type lectins in Jurkat cells**
Jurkat cells were inoculated with lentivirus vectors to express the molecules indicated. Several days later, cells were stained with control antibody (thin line) or with a corresponding antibody (bold line) and analysed by flow cytometry.

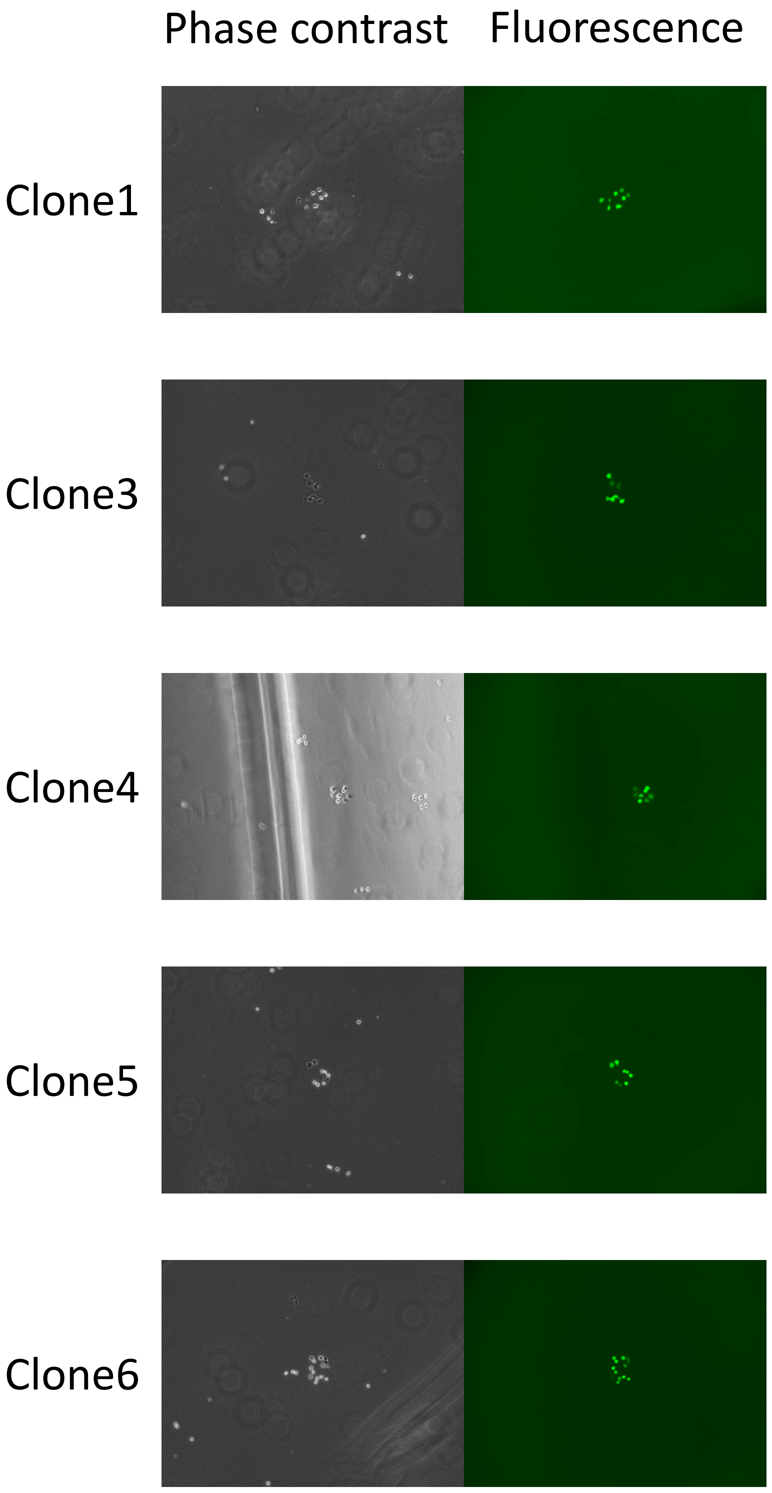


**Supplementary Fig. S4: Human liver cDNA library screening using infectious virus-like particle (iVLP)**

In screening of the human liver cDNA library to identify SFTS virus entry factors, six Jurkat colonies (Clones1–6), which consisted of Venus-expressing cells, were found on a panning dish and five of them (Clones1, 3-6) are shown.
